# Supplementary figures and images for: Identification and characterization of a ubiquitin E3 RING ligase of the Chlamydia-like bacterium Simkania negevensis
Source: PLoS Pathog. 2025 Nov 6;21(11):e1013626. doi: 10.1371/journal.ppat.1013626 (PMC12591485; doi:10.1371/journal.ppat.1013626)

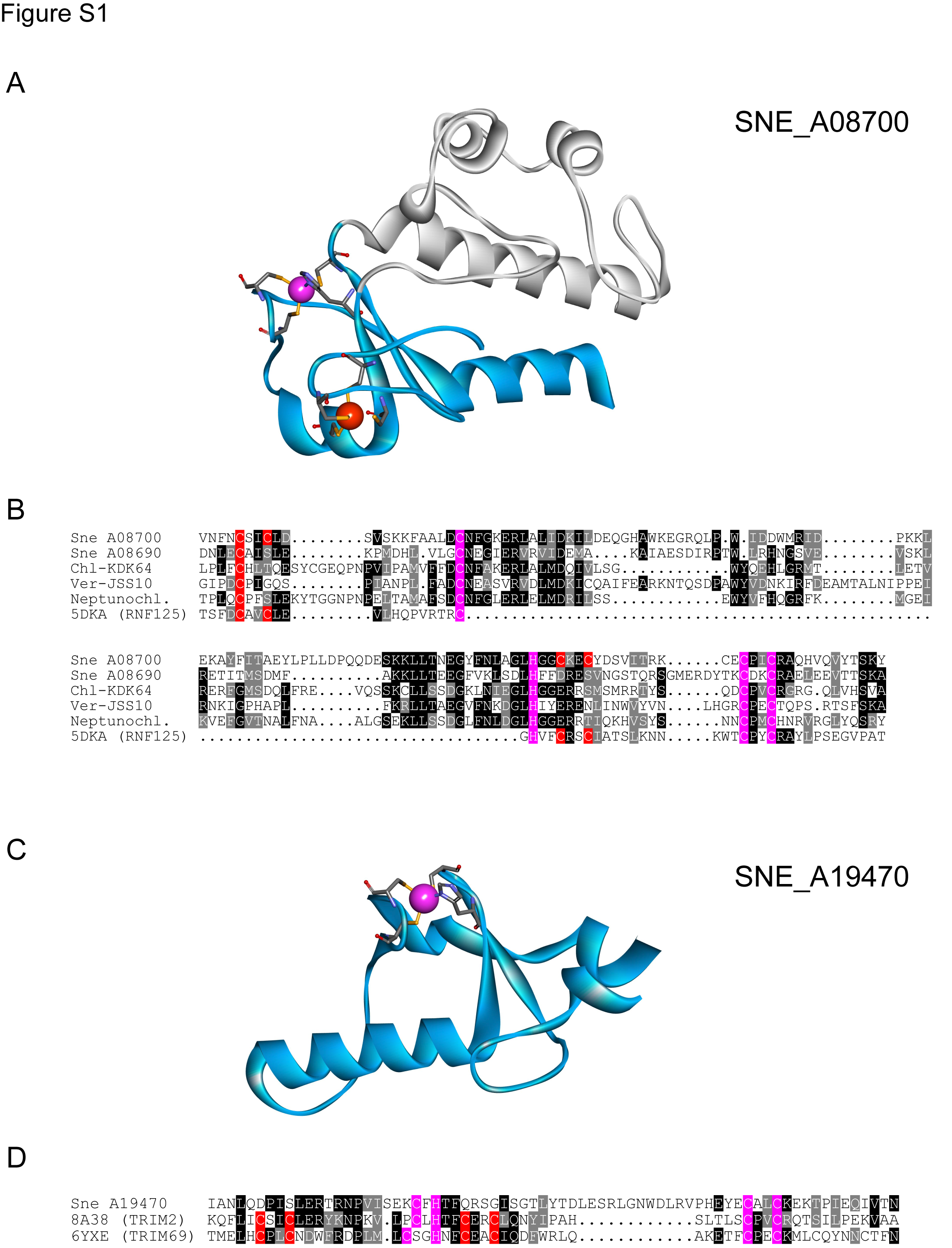

Supplement: S1 Fig — (A) The Alphafold model of SNE_A08700 shows an N-terminal RING-like domain (blue) with a large insertion after the third Zn-coordinating residue (grey). The Zn1 and Zn2 ions coordinated by the RING-like domain are shown in red and magenta, respectively. (B) Multiple alignment of the RING domains of SNE_A08700, SNE_A08690, and some bacterial relatives (Chlr-KDK64: Chlamydiia bacterium, Uniprot: A0A960RSF6; Ver-JSS10: Verrucomicrobiota bacterium, Uniprot: A0A9E0XGC6; Neptunochl: Candidatus Neptunochlamydia sp., RefSeq: WP_316358199) and the sequence of the best DALI hit (pdb:5DKA). Residues invariant or conserved in at least 50% of the sequences are shown on black and grey background, respectively. Residues involved in the coordination of Zn1 and Zn2 are highlighted in red and magenta, respectively. (C) Alphafold model of the RING-like domain of SNE_A19470 shows a divergent RING fold (blue) where only the ligands of the Zn2 ion (magenta) are conserved. (D) Multiple alignment of the RING-like region of SNE_A19470 with the two best DALI hits (pdb:8A38 and pdb:6YXE). Coloring as in B. (TIF) [file ppat.1013626.s001.tif]

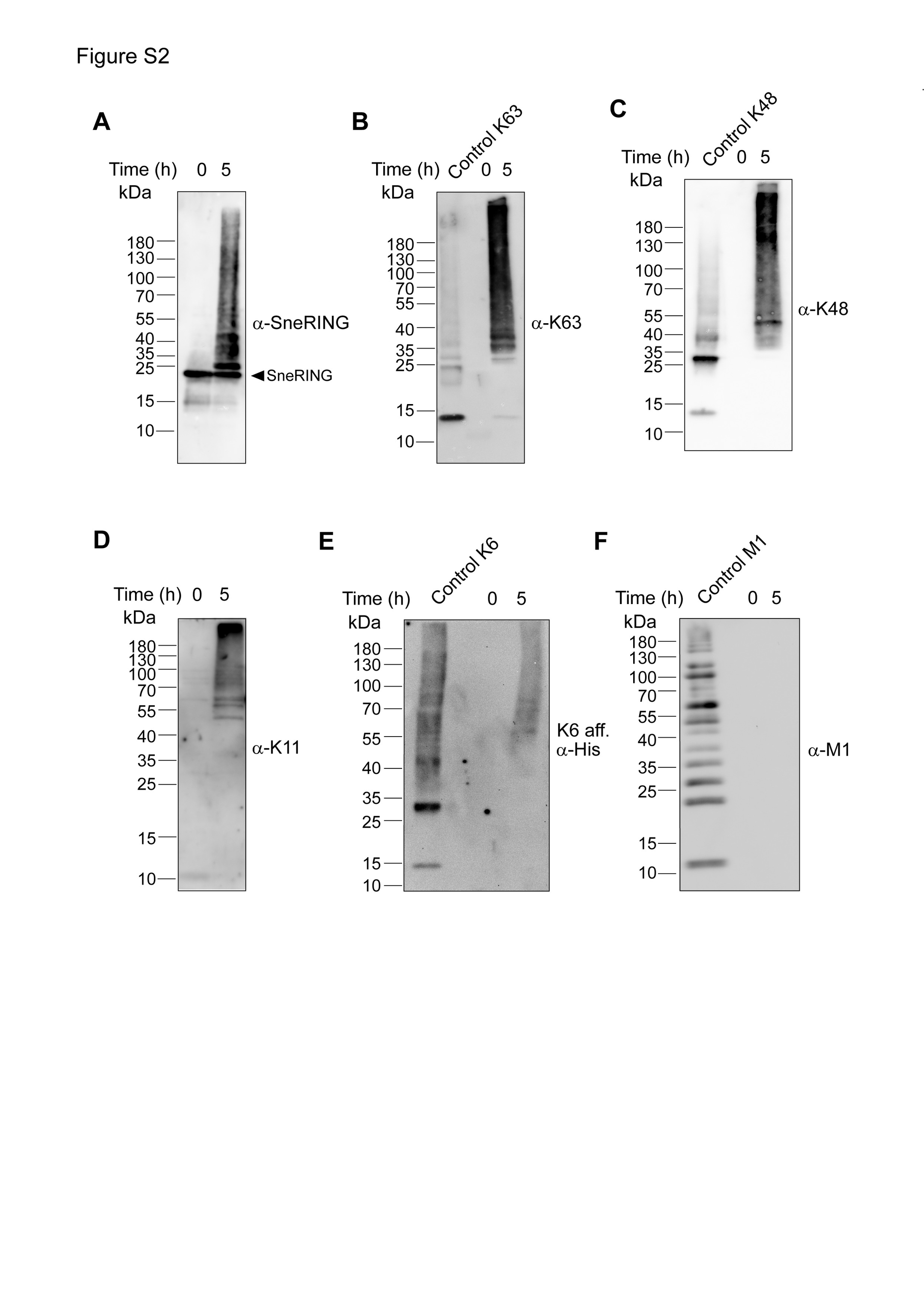

Supplement: S2 Fig — (A-F) In vitro autoubiquitination assay, as in Fig 2A, was performed for 5 h at 37 °C. The reaction was analyzed by SDS-PAGE and western blot, using primary antibodies against (A) SneRING, (B) K63-, (C) K48-, (D) K11-, and (F) M1-linked ubiquitin chains. For the detection of K6-linked ubiquitin chains (E), a His-tagged K6 affimer was used, subsequently detected by His-tag antibody. Positive controls were purchased (K63-, K48-, and K6-linked chains in B, C and E, respectively) or synthesized in vitro (M1-linked ubiquitin chains, F). (TIF) [file ppat.1013626.s002.tif]

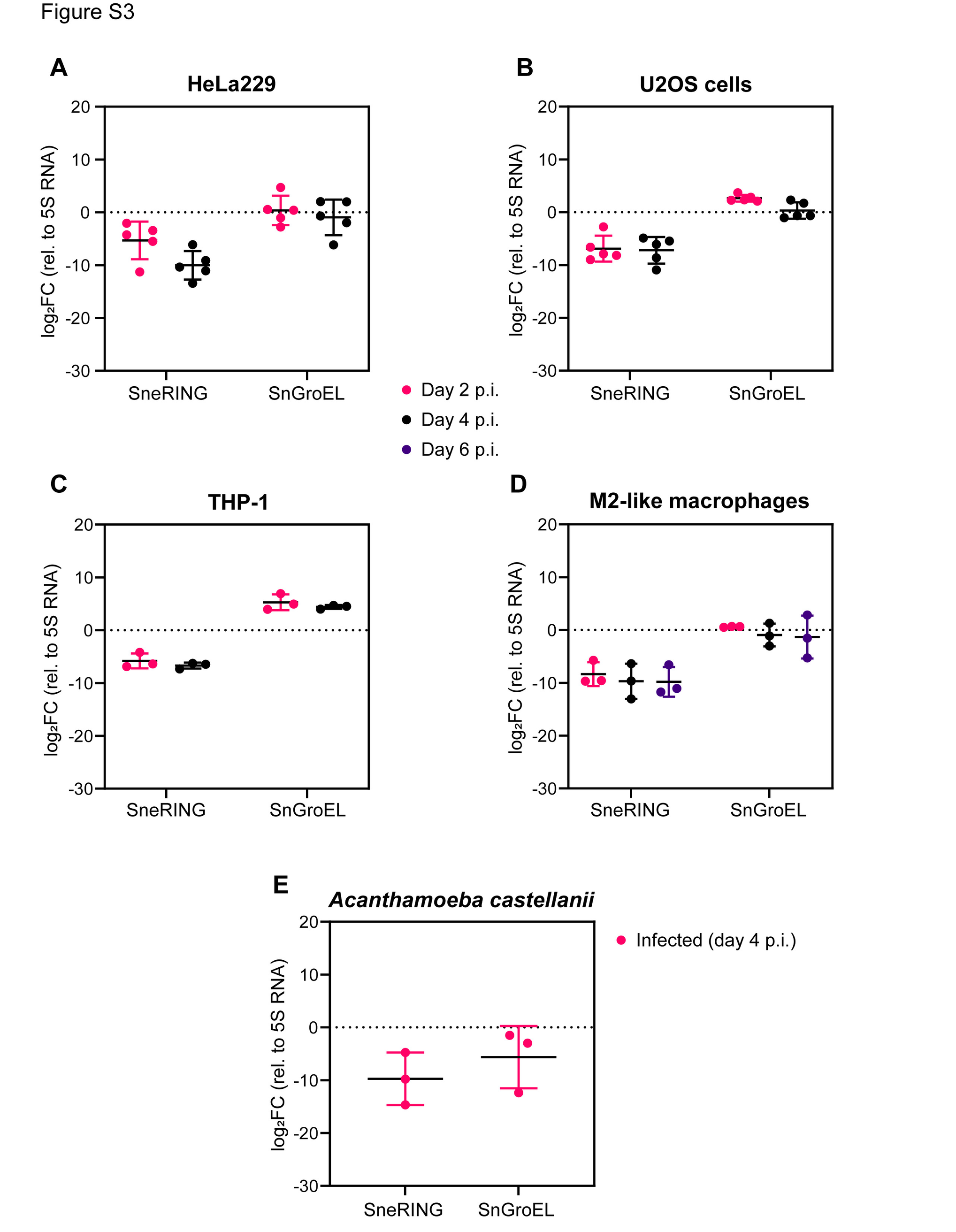

Supplement: S3 Fig — (A, B) HeLa229 or U2OS cells were infected with Sne at an MOI 1, and RNA was isolated on days two and four p.i. (C) THP-1 cells were differentiated into macrophage-like cells using PMA, infected with Sne, and RNA was isolated on days two and four p.i. (D) Primary human “M2”-like macrophages were derived from peripheral blood monocytes (M-CSF/IL-4). After infection with Sne, RNA was isolated on days two, four, and six p.i. (E) A. castellanii was infected with Sne, and four days p.i., RNA was isolated. A modified 2-ΔΔCt method was used to quantify the expression of SneRING and SnGroEL for each time point. ΔCt values were calculated relative to the human reference gene YWHAZ (A, B, C, and D) or 5S RNA of A. castellanii (E). log2 fold change (log2FC/2-ΔΔCt) for each tested Sne gene vs the non-infected control sample was calculated. Expression levels relative to Sne 5S RNA are shown as mean ± SD from independent biological replicates (n = 3 or n = 5). Raw data of the graphs can be found in S2 Table. (TIF) [file ppat.1013626.s003.tif]

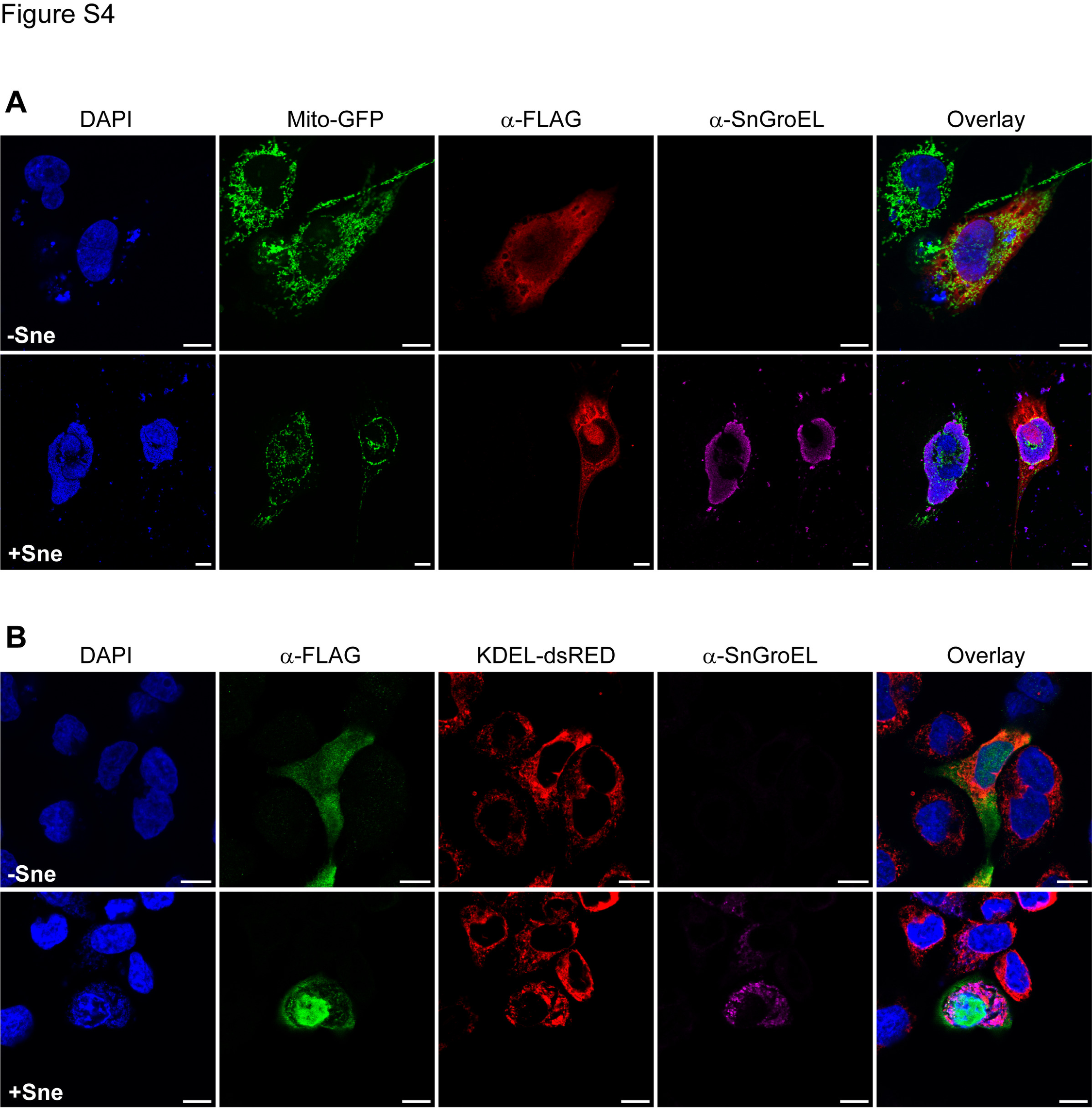

Supplement: S4 Fig — (A) FLAG-tagged version of the SneRING was transfected into HeLa229 cells expressing mitochondria-targeted GFP (Mito-GFP, green channel). 24 h after transfection, the cells were either left uninfected (-Sne) or were infected with Sne at an MOI of 1 (+Sne). On day 3 p.i., the cells were fixed and stained with DAPI (blue channel), and primary antibodies against the FLAG-tag (red channel) and SnGroEL (magenta channel), followed by a fluorophore-coupled secondary antibody. (B) HeLa229 cells stably expressing ER-targeted KDEL-dsRED (red channel) were transfected with a FLAG-tagged version of the SneRING. 24 h post-transfection, one set of samples was infected with Sne for 3 days (+Sne), when they were fixed together with control, non-infected cells (-Sne), and stained using DAPI (blue channel), and antibodies against the FLAG-tag (green channel) and SnGroEL (magenta channel), followed by staining with fluorophore-coupled secondary antibodies. All images were taken using laser confocal scanning microscopy. The scale bar is 10 µm. (TIF) [file ppat.1013626.s004.tif]

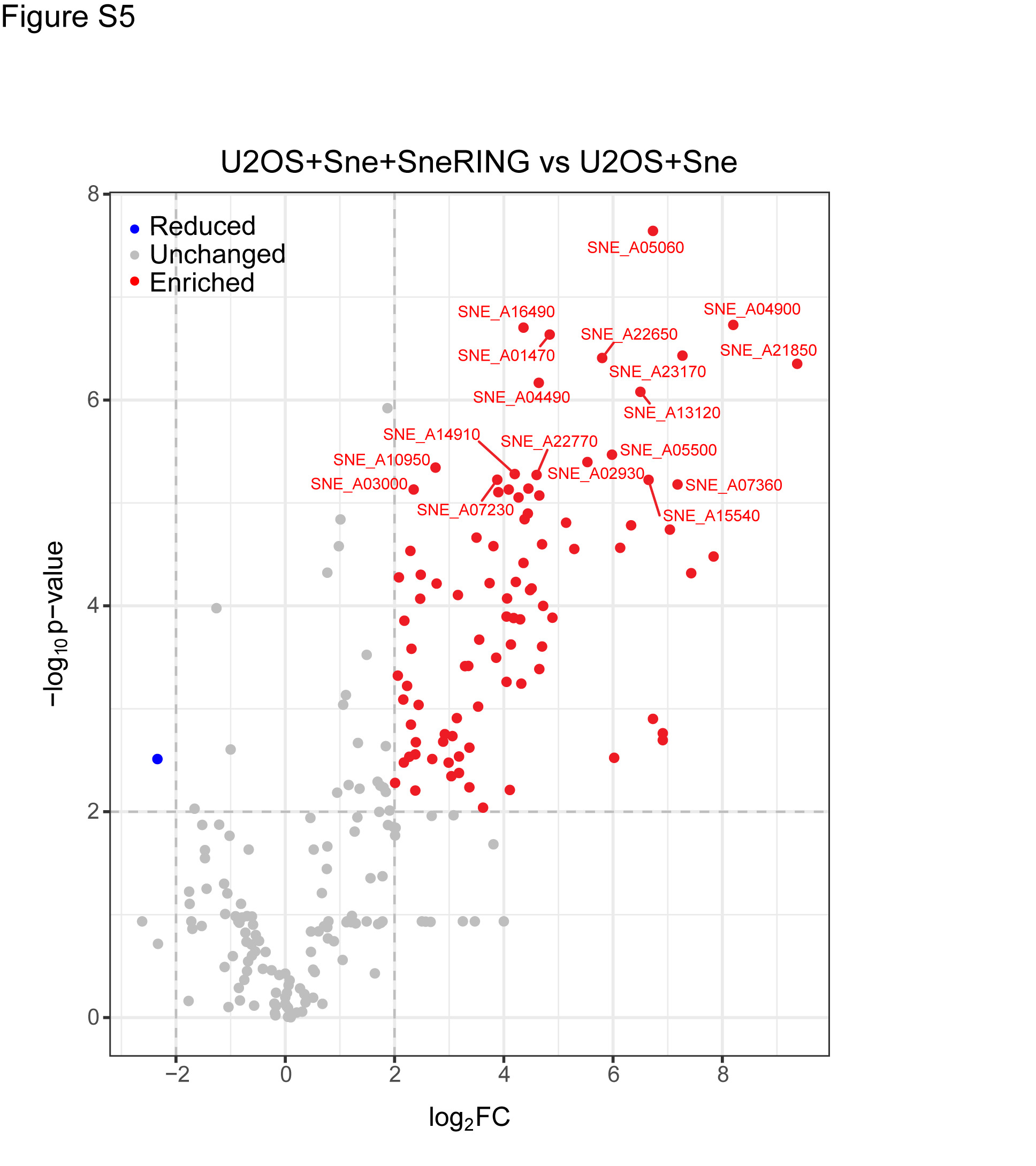

Supplement: S5 Fig — The graph shows identified bacterial proteins from samples described in Fig 5A, with significance (-log10p-value calculated using two-tailed Student’s T-test, n = 3) plotted against the log2 fold change (log2FC) of transfected/infected U2OS cells (U2OS+Sne + SneRING) relative to non-transfected/infected controls (U2OS+Sne). Enriched proteins are presented in red, reduced proteins are labeled in blue, and unchanged proteins are grey. (TIF) [file ppat.1013626.s005.tif]
