# Supplementary material for: Identification and characterization of a ubiquitin E3 RING ligase of the Chlamydia-like bacterium Simkania negevensis
Source: PLoS Pathog. 2025 Nov 6;21(11):e1013626. doi: 10.1371/journal.ppat.1013626 (PMC12591485; doi:10.1371/journal.ppat.1013626)
Supplement: Attachment — Submitted filename: 152741_1_rebuttal_3274536_sqpm7j.docx [file ppat.1013626.s008.docx]

**Manuscript number:** RC-2024-02790

**Corresponding author(s):** Vera, Kozjak-Pavlovic

## 1. General Statements [optional]

***We thank the reviewers for their constructive criticism and the time they took to read and review our manuscript. We will aim to respond to the majority of the points raised by reviewers while offering explanations for the experiments we believe will not contribute to the conclusions of the manuscript or are not possible to perform due to the current experimental limitations.***

## 2. Description of the planned revisions

*Insert here a point-by-point reply that explains what revisions, additional experimentations and analyses are planned to address the points raised by the referees.*

Reviewer #1 comments:

*Simkania negevensis* is a Gram-negative, *Chlamydia*-like bacterium known for possessing an unusually large number of deubiquitinating enzymes (DUBs). It can infect a wide range of host species and has been associated with the development of human pathologies, including respiratory infections as well as inflammatory bowel diseases. Despite this, many aspects of the interaction between the bacterium and host remain unclear. Previous studies have shown that other bacteria such as *Legionella*, *Salmonella*, and *Chlamydia* seem to be able to manipulate the host's ubiquitin system. Therefore, the authors of this study focused on the newly *discovered S. negevensis* ubiquitin E3 RING-ligase (SneRING), its ability to ubiquitinate target substrates and its functional relevance in biological pathways. In this study it was shown, that SneRING to preferentially interact with the E2 enzyme UbcH5b by its interacting key residues S19 and E22 as predicted by structural models from AlphaFold. In vitro autoubiquitination assays with purified proteins confirmed that SneRING is a functional E3 ligase, and a mutation of the interacting residues results in a catalytically inactive form of SneRING. Ubiquitin Chain Restriction assays gave evidence that SneRING is able to generate K6-, K11-, K48-, and K63-linked ubiquitin chains, suggesting that its host cell targets may undergo similar modifications. Additional confocal microscopy suggested that SneRING localizes to mitochondria and the ER, and that its interacting proteins are involved in organelle morphology, respiration, and stress responses. The authors of the study suggested that *S. negevensis* thereby promotes its survival and replication within the host by manipulating ubiquitination pathways within the host, and therefore potentially controlling mitophagy, and managing ER stress.

The conclusions regarding the interaction of SneRING with the E2 enzyme UbcH5b and its catalytic activity are supported by in vitro autoubiquitination data. However, the manuscript lacks in vivo validation using infection models, limiting the broader applicability of the findings. Further studies are required to substantially explore the functional role of SneRING in host-pathogen interactions. The authors of the study should focus on showing that SneRING remains its catalytic activity in infection models and put more emphasis on the validation of potential SneRING target substrates.

***Reply: We agree with the reviewer on this point. Unfortunately, genetic manipulation of* S. negevensis *has so far not been achieved. We can therefore only rely on protein expression in human cells to assess the function and localization of this protein.***

***To perform initial validation of potential SneRING substrates and interacting partners we have or will perform the following experiments:***

- ***Using immunoprecipitation and western blot, we have confirmed the interaction of expressed FLAG-SneRING with several of the targets identified by our interactome analysis (revised Figure 5). We also show that this interaction takes place only in infected cells, confirming our mass spectrometry data and indicating the role of other bacterial factors and/or compartment remodeling by the infection in this process. These experiments will be repeated for consistency.***
- ***We will test if any of the target proteins we currently have antibodies for shows a shift to higher molecular weight or smearing indicative of ubiquitination. This would demonstrate that these proteins are not only interacting with SneRING, but are being ubiquitinated in its presence, too. We will use catalytically inactive SneRING S19R_E22R as a negative control. However, we cannot predict the results of these experiments.***

***More detailed validation of potential SneRING targets would require significant effort and time, and we believe it is beyond the scope of the current manuscript, which focuses on the initial characterization of SneRING.***

Subsequently, the discussion of SneRING's role in modulating host cell processes such as mitophagy and ER stress by manipulating the host ubiquitination pathways should be considered preliminary at this stage due to the lack of experimental validation in in vivo cellular or infection models. The authors should be cautious in suggesting that SneRING controls these processes without underlining the hypothesis with sufficient supporting data.

***Reply: We agree with the reviewer and will modify the abstract/discussion accordingly to limit our speculations and focus on hypotheses where we have confirmatory experimental data.***

While experiments, like additional mass spectrometry analyses and immunoprecipitation of potential substrates, would require additional resources and time, they are feasible and necessary to strengthen the hypothesis in this manuscript. Genetic studies (e.g. knockout/knockdown or knock-in models) will require a more significant time investment but would be important for validating the claims of the manuscript. Additional validation of SneRING targets through methods like Western Blotting and immunofluorescence could be done within a reasonable timeframe.

***Reply: We have performed immunoprecipitation and western blot experiments to confirm several targets of the mass spectrometry data (revised Figure 5). We plan to test if any of the target proteins we currently have antibodies for show a shift to higher molecular weight or smearing indicative of ubiquitination (please see reply to the first point raised by the reviewer).***

The methods for the in vitro experiments are generally well-described. However, the mass spectrometry protocol is lacking important key information, and the statistical analysis is not well-explained and partially missing, making reproducibility difficult. Furthermore, in the methods section some technical questions remain and should be addressed, such as the inclusion of iodoacetamide (IAA) in the lysis buffer, which could cause artifacts mimicking ubiquitination signature in mass spectrometry analysis, especially since it remains unquenched***.***

***Reply: We agree with the reviewer and have amended the material and method section, as well as figure legends to include the lacking data, especially regarding statistical analysis of the mass spectrometry results of the interaction partners of SneRING, which was the standardly used two-tailed Student’s T-test (n=3).***

***Regarding the usage of IAA, it indeed produces lysine adducts that mimic the diglycine tag. However, as it is written in the present material and methods section, IAA was used only in the experiment where we identified the interaction partners of SneRING. The reason for this was that IAA alkylates catalytic cysteine residues of deubiquitinating enzymes and therefore inhibits them. The idea behind it was to prevent the deubiquitination of potential SneRING substrates by cellular DUBs. In this case, however, since we did not focus on detecting ubiquitination signatures on these proteins, the presence of IAA should not influence the results.***

***For downstream mass spectrometry analysis, we used chloroacetamide (CAA) as an alkylating agent. CAA does not produce such artifacts (as reported by Nielsen et al, 2008,* DOI:** [**10.1038/nmeth0608-459**](https://doi.org/10.1038/nmeth0608-459) ***).***

Many experiments lack adequate replication. For example, the Western blot data is not replicated (n=3 or more). The study would benefit from additional experimental replicates to ensure the robustness of the findings.

***Reply: We fully agree with the reviewer about the importance of replicating experiments to ensure reproducibility and confirm the finding but are unsure about what made the reviewer conclude that many of our experiments lack adequate replication. For the majority of the western blot data, the experiments have been replicated in duplicate, and specific time points have been replicated at least in triplicate if not more:***

***- The autoubiquitination assays in Figure 2 are all independent experiments that all show reproducibly SneRING autoubiquitination in the presence of UbcH5b and UBE2W***

***- The experiment in Figure 2B has also been repeated in triplicate.***

***- Western blot analysis of the autoubiquitination assay at 5 h with different antibodies in Figure S2 has been repeated to duplicate the western blot. However, the same information about the type of ubiquitin chains has been provided by the UbiCRest assay and mass spectrometry in Figure 3. Therefore, we are of the opinion that a third western blot in this case would not significantly add to the conclusions of the manuscript.***

***- Cell fractionation experiments shown in Figure 4 have been performed in two different cell lines (Hek293T and U2OS), but we show only the results for U2OS cells. We will repeat the experiment in U2OS cells to obtain the third replicate.***

***- We will perform additional experiments with mitochondrial isolation and swelling to have the data from at least two different cell lines in duplicate.***

Regarding the immunofluorescent images more images should be provided, especially since it is unclear how many cells were imaged.

***- Microscopy experiments have been performed numerous times, in different cell lines (Hek293T, HeLa, U2OS). We have made dozens of images and will include better images in Figure 4, but since these results are of a descriptive nature, and are supported by cell fractionation and western blot, we believe that showing more images will not contribute to the conclusions of the manuscript.***

Minor comments:

The introduction requires clarification and some reorganization for better structure. The manuscript would benefit from a more consistent and thorough citation of prior studies, especially in the introduction (e.g. lines 73-74).

***Reply: We will include additional citations regarding* S. negevensis *and prior studies on these bacteria. We will likewise streamline and shorten the introduction, as also requested by reviewer 3.***

More context is needed to explain how *Simkania negevensis* contributes to human diseases, as the current statements about its pathogenic role are unclear or contradictory (e.g. lines 56-71).

***Reply: We will add additional information, although, in our opinion, the pathogenicity status of these bacteria is not important for the conclusions about SneRING presented in this manuscript.***

Figure 2: Some figures need clearer labels and additional controls. For instance, the absence of a loading control in Figure 2 should be addressed (e.g. adding Coomassie-staining), and clearer experimental setups are needed such as additional arrows indicating mono-ubiquitination, di- or poly-ubiquitination, or free ubiquitin should be added.

***Reply: As per reviewer’s suggestion we have revised Figure 2 to indicate mono-, di- and poly-ubiquitin. We are including a Coomassie staining of Figure 2B that we have for reviewer information. We believe that for the rest of the western blots in this figure the signal for the mono-ubiquitin, though not ideal, can serve as a solid loading control. However, we will also include an additional loading control in the form of SneRING signal, similar to what we have already included in the revised Figure 3A. The SneRING antibody that we have raised against the full-length protein recognizes the purified protein but unfortunately did not detect the protein in the lysates of infected cells or in microscopy studies.***

Additional in vitro autoubiquitination assays utilizing FLAG-tagged SneRING, with an anti-FLAG antibody should be performed. This approach would also demonstrate that FLAG-tagged SneRING retains its catalytic activity.

***Reply: We can perform the autoubiquitination assay using FLAG-tagged SneRING to show the functionality of the tagged protein, but this will take additional time and effort we believe is not entirely justified. Our reasoning for this is the following:***

- ***If by staining this reaction with an anti-FLAG antibody, the reviewer wishes us to show that it is SneRING itself that is being ubiquitinated in the autoubiquitination reaction, we showed this by using an anti-SneRING antibody that detects the purified protein (please see revised Figure S2A). We have added the following sentence to the manuscript (lines 209, 210): “The resulting signal represented an autoubiquitinated SneRING because a similar pattern could be observed when the reaction was analyzed using SneRING antibodies (Fig S2A).”***
- ***If the reviewer wishes us to show that the presence of a FLAG-tag at the amino terminus of the protein does not interfere with its function, it is important to note that the purified SneRING we used in the study for all in vitro assays already contains the addition of two amino acids (glycine and proline) at the amino-terminus due to the cloning, expression, and purification procedure. We have included this information in the Material and Methods section. The additional six residues present in the FLAG-tag will highly likely not affect the functionality of the RING ligase. Indeed, often a much larger tag, such as GST tag is added to RING domain-containing proteins used in the autoubiquitination assay without affecting their functionality, especially if the proteins themselves are small and lack lysine residues (one example is found in Kikker et al, 2003,*** DOI: [10.1074/jbc.M307453200](https://doi.org/10.1074/jbc.m307453200) ***).***

***We hope the reviewer will accept this argumentation as valid, in addition to the data we show in revised Figure S2A.***

Figure 3B) What is the difference between the "without DUB" and "5 hours control" conditions? Why is there no sample in lane 3? The treatment with CEZANNE appears different in Figure 3A and 3B-why is that?

***Reply: The reaction in the lane “5 hours control” is the same sample as in the lane “without DUB”. The latter has been additionally incubated in the same buffer and for the same time as the DUB reactions. The line 3 is empty to separate the assay from the controls. We now removed the first two lanes of the blot from the image to avoid confusion. The treatment with CEZANNE looks different because it is a different reaction analyzed on a different gel, with the first western blot being recorded with a longer exposure time and appearing darker. However, the pattern of the bands is comparable, with the loss of higher poly-ubiquitin bands.***

Figure 3C): The data presented in Figure 3C contradict the text. In the text, it is stated that the mass spectrometry data predominantly show K11 and K63 as enriched, with only some signal for K6 and K48-modified residues. However, the difference in log10(intensity) is only around 5%.

***Reply: We understand this issue; however, Figure 3C uses a logarithmic scale. The observed difference between K11/K63 and K6 is 0.68 log10 units, which corresponds to a factor of 4.7. Thus, K11 and K63 chain intensities are not 5% above K6 intensities but almost 5-fold stronger. We have modified the text and rephrased “Only some signal for K6” into “about 5-fold lower signal detected for K6” to be more precise in our claims.***

Figure 4A): The FLAG-tagged antibody seems to be diffused/has a high background throughout the cell, making it difficult to interpret.

***Reply: We will provide better images for this figure; however, the diffused signal has been already addressed in the text and is one of the reasons why we resorted to cell fractionation, which offers more reliable information in comparison to mostly descriptive microscopy images.***

If feasible, as an additional experiment, FLAG-tagged SneRING S19R_E22R (catalytically inactive) should be transfected and compared to wild-type SneRING. In addition to that, ubiquitin-chain specific antibody could be utilized for immunofluorescence studies.

***Reply: We have already transfected cells with SneRING S19R_E22R but did not observe any difference of distribution in comparison to the active form of the enzyme. We can include these images if required. We have also already performed microscopy studies using K63 ubiquitin chain-specific antibodies that work well in immunofluorescent staining; however, they are not very conclusive and this is why we have not included them in the current manuscript.***

Figure 4C): Why were Mic60 and Calnexin not in the control and total lysate fraction detected?

***Reply: Mic60 and Calnexin are detected in the control and total lysate fraction, but the signal is very weak. This is because the control and total samples come from whole cells, in which Mic60 and Calnexin are not as abundant as tubulin or GAPDH, whereas purified crude mitochondria and light membrane fractions are much more concentrated samples of compartments where Mic60 and Calnexin proteins are usually found. Therefore, the signal there is much stronger (in comparison, GAPDH shows reverse behavior, due to this being an abundant cytosolic protein).***

Figure 4D): Why was TIM44 degraded upon swelling and Proteinase K treatment? And why was CPOX still detected under the PK+1% Triton condition, as everything should have been degraded in this treatment?

***Reply: Tim44 is a protein associated with the matrix side of the inner mitochondrial membrane. During swelling, mitochondria are vigorously pipetted in a hypotonic buffer to enhance the rupture of the outer membrane. This can partially also damage the inner membrane, making a portion of the Tim44 accessible to the proteinase K. Therefore, we see a partial degradation of Tim44.***

***CPOX is a tightly folded protein of the mitochondrial intermembrane space. Most of it is lost upon swelling which is reflected in the reduced signal in the + swelling lanes. The addition of the 1% triton coupled with the proteinase K treatment enables proteinase K to largely digest this protein due to the permeabilization of membranes by triton detergent; however, a tightly-folded core remains non-digested (please observe the size shift of the signal). We observe this repeatedly in similar experiments (for comparison, please see the Fig. 2d in publication*** DOI: [10.1016/j.jmb.2013.12.001](https://doi.org/10.1016/j.jmb.2013.12.001) ***); similar is observed for another tightly-packed mitochondrial protein Hsp60 (please see Fig 2b in*** [DOI: [10.1371/journal.pone.0218303](https://doi.org/10.1371/journal.pone.0218303)](https://doi.org/10.1371/journal.pone.0218303) ***).***

Figure 4E): The author mentions that a small portion of SneRING appears to be membrane-integral, but this claim should be explained more thoroughly, as the Western blot data does not show any strong difference in the carbonate extraction assay.

***Reply: We will include an additional explanation. The signal for FLAG is stronger in the supernatant fraction, showing that more protein is in the soluble fraction, but it is difficult to precisely quantify the amount.***

There are inconsistencies in the transfection times, which vary between 24, 36, and 72 hours. A clear explanation for these variations is needed.

***Reply: The observed different time points of transfection are the consequence of some of the transfected samples being infected afterward. We will modify the materials and methods section to make this clearer.***

***In general, all samples for mass spectrometry and the immunofluorescence microscopy were transfected for 24 h prior to infection, then incubated for another 48 hours, which totals in 72 h from the moment of transfection.***

***Since 72 h is not necessary for a good expression of SneRING in transfected cells, we used shorter expression times (36 h and 48 h) for fractionation experiments in Figure 4. Considering that the amount of expressed FLAG-SneRING is comparable in these samples, we believe that different expression times do not influence the results of the experiments.***

***Of note – the cells used in the figure 4D and 4E are Hek293T and not U2OS cells as we initially wrote and we have changed the figure legend accordingly. We plan to repeat these experiments with mitochondria isolated from U2OS cells for additional control.***

There are formatting issues, such as inconsistent use of units and unclear figure legends, that need to be addressed. The abbreviation "pi" for "post-infection" is problematic, as "pi" is commonly used as a mathematical constant. A more suitable abbreviation should be chosen.

***Reply: We will check the manuscript again for any inconsistencies. In microbiology the standard abbreviation for post infection is pi. Since we understand that this might be confusing to the people outside of the field, we exchanged it for p.i. throughout the text.***

The identification of SneRING as a novel E3 RING ligase in *Simkania negevensis* is a valuable contribution to the understanding of bacterial manipulation of the host's ubiquitination system. Ubiquitin ligases play crucial roles in regulating cellular processes, and the conclusions from this manuscript could contribute to the understanding of how bacteria may manipulate host cell pathways to promote infection. The findings of this study could lead up to new prospects regarding therapeutic interventions targeting bacterial manipulation of host ubiquitination processes.
Previous studies have shown that other bacteria, such as *Legionella* and *Salmonella*, manipulate the host's ubiquitination system, but the role of ubiquitin E3 ligases in *Simkania negevensis* is largely unexplored. This study extends these findings by identifying a novel E3 ligase, SneRING, and providing initial insights into its function. However, the paper would benefit from a more comprehensive discussion of related studies and how SneRING fits within the broader field of bacterial E3 ligases.

***Reply: We thank the reviewer for the supportive assessment of the manuscript. In our discussion, we have addressed other known bacterial ubiquitin ligases and their potential roles in infection; however, these ligases are mostly of HECT type and therefore not entirely comparable to SneRING. We will try to provide a more comprehensive discussion on this point in the revised version of the manuscript.***

Reviewer #2 comments:

The authors investigate the *Chlamydia*-like bacterium *Simkania negevensis* for the presence of RING domain ubiquitin E3 ligases as several DUBs had been identified in *Simkania* recently. The authors now identify SneRING based on sequence homology and Alphafold predictions as a RING domain containing protein and demonstrate E3 activity of recombinant protein in vitro. SneRING preferentially works with UBCH5B and produces a variety of linkage types, including K6, K11, K48 and K63 linked chains. Expression of SneRING reveals a diffuse cytoplasmic distribution of the protein although in follow up experiments the authors concentrate on a potential association with mitochondria. Immunoprecipitation of overexpressed SneRING led to the identification of possible host-derived interaction partners.

This work presents a first analysis of RING E3 ligases in *Simkania*, which could serve as a starting point for future investigations regarding the role of bacterial E3 ligases in manipulating host cells. The manuscript will therefore be of interest to microbiologists studying host-*Chlamydia* interactions or possibly more generally host-pathogen interactions. The identification of SneRING as a Ub E3 ligase is convincing as the catalytic activity is well demonstrated, including through point mutations in the proposed E2 interacting surface of the RING domain. The association of SneRING with mitochondria seems less convincing. To my eye, the majority of the protein appears diffuse in the cytosol and not associated with mitochondria. While the authors provide some biochemical evidence, I am concerned that overexpression and the possibility of cytosolic contamination of mitochondrial preps (including possibly SneRING aggregates) may have obscured the real picture.

***Reply: We agree with the reviewer and will therefore discuss these results with more caution. What speaks in favor of mitochondrial localization is the degradation of the protein upon swelling and PK treatment of mitochondria (usually, aggregated proteins contaminating the mitochondrial preparations would not demonstrate such behavior). In addition, we present additional immunoprecipitation experiments showing interaction with a mitochondrial inner membrane protein Mic60 taking place upon infection only, which strongly supports mass spectrometry data (revised Figure 5) and will modify our conclusions and discussion accordingly.***

The identification of cellular interactors may also be helpful for future studies, although the immediate relevance of any of the hits is not clear.

***Reply: We can only speculate on the relevance of the hits; however interestingly, we could confirm that interactions with at least some of the hits take place in an overexpression scenario but only in infected cells, indicating the role of other bacterial proteins, or the necessity for the reorganization of cellular compartments during infection for this interaction to take place.***

***In addition, we are in possession of the knockdown cell lines for some of the putative interactors, such as Mic60 (IMMT) and Sam50, where we have often observed that the protein knockdown and disturbance of mitochondrial morphology affects the development of Simkanial inclusion. These experiments, however, have not been sufficiently repeated and some experimental details have still not been clarified (one problem being the sensitivity of Simkania to anhydrous tetracycline, which is used to induce the knockdown). We therefore do not want to include these results in the current manuscript but will continue to analyze the relationship between Simkania, mitochondria, and ER.***

Reviewer #3 comments:

The manuscript by Hörner et al reports an E3 ubiquitin ligase from the bacterial pathogen *Simkania negevensis*, the authors started the project by bioinformatic analysis of proteins coded for by *S. negevensis*, and found that SNE_A12920 (later designated as SneRING) has detectable similarity with members of the RING family E3 ligases. Further biochemical experiments demonstrate that SneRING indeed has ligase activity by working with UbcH5b or UBE2T. They also performed experiments to determine the cellular localication of the protein and its potential targets. Overall, the assignment of an E3 ligase activity to SneRING is supported by strong results but other other parts of the manuscript are somewhat confusing (the rationale for some of the experiments is not clear) and some conclusions need to be strengthened by additional experiments.

***Reply: We thank the reviewer for the positive assessment of the manuscript. We hope that the additional data we will show (revised Figure 5) explains the rationale for some of the experiments and strengthens in part the conclusions of the manuscript.***

Specific comments:

In experiments attempting to identify the host proteins targeted by SneRING, it is not clear why samples from *S. negevensis*-infected cells were included. Do the authors suggest that SneRING targets *S. negevensis* proteins (potentially other virulence factors injected into infected cells?). This possibility is there but the likelihood is low because such proteins often is injected at extremely low quantity. The identification of bacterial proteins is very confusing.

***Reply: We reasoned that it is possible that additional* S. negevensis *proteins are required for targeting or functionality of the SneRING, or that the reorganization of the cellular internal membrane compartments contributes to the localization of SneRING. This is why we included the samples from* S. negevensis*-infected cells in the pulldown experiments. Indeed, in the revised Fig. 5, we show the pulldown experiments where we observe that the interaction with selected host-cell targets is enhanced by infection, supporting the mass spectrometry results and an idea that additional* S. negevensis *proteins are required for targeting or functionality of the SneRING. Such possible interactors could be among the bacterial proteins we show in Fig. S5 and this is why we have decided to show those results in addition to the list of the putative host-cell interacting proteins.***

As an E3 ligase, SneRING should induce ubiquitination on its targets, yet none of the identified candidates was examined for SneRING-induced ubiquitination, which makes the results in Fig. 5 irrelevant.

***Reply: What we currently show in Fig. 5 of the manuscript are proteins we consider to be interactors and substrate candidates. Whereas we agree that it would be nicer if we could have shown that these are real substrates of the SneRING, we do not agree with the reviewer that these results are irrelevant, especially since we could confirm the interaction of SneRING with at least some of them (revised Fig. 5).***

***During the revision of the manuscript, we will use the antibodies we have against some of the SneRING interactors and substrate candidates (Mic60, Erlin2, CKAP4, and Prohibitin) to test if upon overexpression of the active and inactive SneRING we can see that the signal of the protein is reduced and/or shows ubiquitination bands/smears when the active form of SneRING is present, which would indicate that it is a substrate of SneRING. This will have to be done in infected cells, since our data so far supports the idea that infection is necessary for SneRING localization or full functionality. However, we have no guarantee that this experiment will work, and one may argue that even if we observe such an effect, it could be indirect. Further studies in this direction will require time and effort that we will invest in the future, but we feel are beyond the scope of the present study.***

The Introduction and Discussion are too lengthy, please consider making them more concise.

***Reply: We will rewrite the Introduction and Discussion to accommodate the comments of reviewers 1 and 2 as well.***

## 3. Description of the revisions that have already been incorporated in the transferred manuscript

*Please insert a point-by-point reply describing the revisions that were already carried out and included in the transferred manuscript. If no revisions have been carried out yet, please leave this section empty.*

Reviewer #1 comments:

The authors of the study should focus on showing that SneRING remains its catalytic activity in infection models and put more emphasis on the validation of potential SneRING target substrates.

***Reply: Using immunoprecipitation and western blot, we have confirmed the interaction of expressed FLAG-SneRING with several of the targets identified by our interactome analysis (revised Figure 5).***

However, the mass spectrometry protocol is lacking important key information, and the statistical analysis is not well-explained and partially missing, making reproducibility difficult.

***Reply: We have amended the Material and Method section, as well as figure legends to include the lacking data, especially regarding statistical analysis of the mass spectrometry results of the interaction partners of SneRING.***

Furthermore, the statistical analysis should be more detailed and include more than just p-values, such as a clear explanation of the statistical tests used and the number of biological replicates.

***Reply: The statistical analysis has only been performed on mass spectrometry data of the interaction partners of SneRING (Figure 5). We included the necessary information in the text and the figure legends.***

Figure 1: The AlphaFold predictions in Figures 1B and 1C appear excessively large, and the space could be better utilized by adding more bioinformatics information. Figure 1C should focus on predicting the entire protein structure, not just the RING domain. The predicted contact residues (S19 and E22) should be corrected in the text, as the order is consistently reversed.

***Reply: We have revised Figure 1 and reduced the size of the Figure 1B and 1C but are unsure what more bioinformatics information we should include that we have not already provided in the text and supplementary data. Since the complete protein is already shown in Figure 1B, we believe that Figure 1C should stay as it is, since it focuses on the SneRING:E2 contact. The text has been corrected according to the reviewer’s suggestion.***

Figure 3A): The concentration of the added DUB protein should be included in the figure legend. Why was not a K33-specific DUB tested?

***Reply: We have added the concentration of the DUBs in the figure legend and in the revised Figure 3B. There is no K33-specific DUB that we know of (OTUD2, which could be used, cleaves also K11-, K27-, and K29-chains).***

Figure 3C): The data presented in Figure 3C contradict the text. In the text, it is stated that the mass spectrometry data predominantly show K11 and K63 as enriched, with only some signal for K6 and K48-modifed residues. However, the difference in log10(intensity) is only around 5%.

***Reply: we have changed the text into: “with about 5-fold lower signal detected for K6- and K48-modified peptides (Fig 3C).”***

Figure 3D): The text lacks an explanation for Figure 3D.

***Reply: We have corrected this.***

Figure 4B): The immunoprecipitation experiment should specify the amount of transfected FLAG-tagged SneRING plasmid.

***Reply: We included this information in the revised manuscript text. We used 10 µg of plasmid for a 10 cm dish initially seeded with 6x10^5^ cells.***

Figure 5): The volcano plots need revision, including colour adjustments to match standard conventions (red for enriched, blue for reduced). All protein names should be clearly readable and should be clearly labelled to its respective data point. The statistical analysis should be explained more thoroughly, and the number of biological replicates should be stated.

***Reply: We changed the coloring in the volcano plots (revised Figures 5 and S5) and added the information to figure legends. Regarding the visibility of the protein names, we removed some names to make the rest more visible. In addition, the manuscript contains Table S1, which lists all the proteins and supports the information presented in the figures.***

The abbreviation "pi" for "post-infection" is problematic, as "pi" is commonly used as a mathematical constant. A more suitable abbreviation should be chosen.

***Reply: In microbiology, pi is commonly used as an abbreviation for “post-infection”. However, we understand this might lead to confusion and have therefore exchanged pi for p.i. throughout the text.***

Reviewer #3 minor points:

Need to define "ER" in the abstract.
Line 65 "extent".

Line 71, would "within a compartment called "Simkania-containing vacuole (SnCV)" be better since ScCV was first introduced here.

Line 130, the ref (PMID: 18284575) for Legionella LubX should be cited.

***Reply: We have implemented all the suggestions and indicated the changes in the text (yellow highlight).***

## 4. Description of analyses that authors prefer not to carry out

Reviewer #1 comments:

The authors of the study should focus on showing that SneRING remains its catalytic activity in infection models and put more emphasis on the validation of potential SneRING target substrates.

***Reply: More detailed validation of potential SneRING targets would require significant effort and time, and we believe it is beyond the scope of the current manuscript, which focuses on the initial characterization of SneRING.***

Additional experiments are essential to validate the biological relevance of the findings. The study primarily relies on in vitro autoubiquitination assays and structural predictions, which do not provide sufficient evidence of the functional relevance of SneRING in a biological context. Genetic study, such as knockout/knockdown or knock-in models, would provide essential in vivo validation. Additionally, more comprehensive mass spectrometry analysis could identify specific substrates of SneRING, which would require subsequent validation processes.

***Reply: Whereas we agree with these points, we are limited by the system we work with, which does not allow us to create deletion mutants of SneRING in* S. negevensis*. Manipulation of putative host cell target genes is possible – however it is coupled with a significant work, which we plan for the future studies and future manuscripts. The same applies to additional mass spectrometry analysis and further validation of the specific targets.***

While experiments, like additional mass spectrometry analyses and immunoprecipitation of potential substrates, would require additional resources and time, they are feasible and necessary to strengthen the hypothesis in this manuscript. Genetic studies (e.g. knockout/knockdown or knock-in models) will require a more significant time investment but would be important for validating the claims of the manuscript. Additional validation of SneRING targets through methods like Western Blotting and immunofluorescence could be done within a reasonable timeframe.

***Reply: We have performed immunoprecipitation and western blot experiments to confirm several targets of the mass spectrometry data (revised Figure 5). The mass spectrometry analysis we show in the manuscript is the result of a triplicate of independent experiments, and we believe that the repetition of these experiments would not bring novel insights. Genetic studies involving genetic manipulation of* S. negevensis *are currently not possible to perform. Further analysis (knockout/knockdown or knock-in models of host cell genes) will be performed in the future and hopefully contribute to further characterization of SneRING and its mode of function – we believe, however, that they are beyond the scope of the current manuscript. From the technical point of view, due to the dispersed signal of the SneRING, immunofluorescence and microscopy will, in our opinion, hardly bring new information about the SneRING localization or its interaction partners.***

An additional control for the single mutations would be important (S19R and E22R).

***Reply: The aim of mutating these residues was to obtain a catalytically inactive version of the protein and to prove that SneRING uses the same E2-binding interface as other RING ligases. Since our experiments with the double mutant show that this seems to be the case, we do not see the benefit of creating single mutant versions of the SneRING.***

Figure 3A) Why was not a K33-specific DUB tested?

***Reply: There is no K33-specific DUB (OTUD2, which could be used, cleaves also K11-, K27-, and K29-linked chains). For more information about DUB specificities, please refer to the following publications*** DOI: [10.1038/nprot.2015.018](https://doi.org/10.1038/nprot.2015.018) ***and*** DOI: [10.1016/j.cell.2013.05.046](https://doi.org/10.1016/j.cell.2013.05.046) ***.***

Reviewer #3 comments:

To determine the chain types induced by SneRING (Fig. 3), the authors need to use ubiquitin variants that retain only one lysine for the biochemical assays. Information derived from experiments using DUBs is not sufficient to support the conclusion as many DUBs are not specific.

***Reply: We agree that using the ubiquitin variants with only one unmutated lysine would support the determined chain specificity of SneRING. However, The DUBs chosen for the UbiCRest assay were selected for their high linkage specificity (as described in the publication by Hospenthal et al,*** DOI: [10.1038/nprot.2015.018](https://doi.org/10.1038/nprot.2015.018)***). Therefore, problems with cross-reactivity are not to be expected. Moreover, the chain types identified by UbiCRest are additionally supported by our mass spectrometry data (Fig. 3C and D) and the use of chain-specific antibodies (Fig. S2). We therefore feel confident that our chain assignment is correct and do not believe that the experiment using ubiquitin variants would significantly contribute to or influence the conclusions of the manuscript.***
