# Supplementary material for: Identification and characterization of a ubiquitin E3 RING ligase of the Chlamydia-like bacterium Simkania negevensis
Source: PLoS Pathog. 2025 Nov 6;21(11):e1013626. doi: 10.1371/journal.ppat.1013626 (PMC12591485; doi:10.1371/journal.ppat.1013626)
Supplement: Attachment — Submitted filename: Response to Reviewers_2.docx [file ppat.1013626.s010.docx]

**Response to Reviewers’ comments**

**Manuscript number:** PPATHOGENS-D-25-00279

**Corresponding author:** Vera Kozjak-Pavlovic

Reviewer #1:

T*his reviewer fully understands the fact that genetic manipulation of* S. negevensis *currently is not possible, but this is NOT the question in focus. The question of relevance here is whether any of the interacting proteins identified by the authors is directly ubiquitinated by SneRING. The key experiment is to use biochemical reactions to show that one or more of these proteins are ubiquitinated by SneRING. If recombinant proteins from* E. coli *cannot be detectably modified by the bacterial E3 in such assays, the authors may obtain the candidate proteins from mammalian cells by transfection and then immunoprecipitation. If these proteins cannot be detectably ubiquitinated by SneRING, the identification of these proteins as the binding targets is not relevant to its activity as an E3.*

**Reply:** To address this, we have aimed to purify CKAP4 and Mitofilin (IMMT) as recombinant proteins expressed in *E. coli*. However, we were not successful. Therefore, we acquired commercially available proteins His-CKAP4 and GST-Mitofilin. The subsequent *in vitro* ubiquitination reactions showed that CKAP4 can be ubiquitinated in the presence of SneRING ligase. In comparison, purified GST was not ubiquitinated by SneRING, indicating a certain substrate specificity of the SneRING. We are showing this data in the new Figure 6 and have modified the manuscript text to include this information.

The results concerning GST-Mitofilin were not as clear. We did observe the appearance of a higher molecular weight Mitofilin signal in reactions containing SneRING, but due to the large size of the construct and relatively low amounts of the purified protein that we had available, we were unable to further analyze these signals. Therefore, we have decided not to include this image in the manuscript but are including it for reviewer’s information.

Interestingly, we repeatedly observed that the autoubiquitination of SneRING was significantly reduced in the presence of a substrate such as CKAP4 or Mitofilin, whereas the presence of GST did not have a similar effect. This might further indicate specific interaction of SneRING with potential substrates.

We hope that this sufficiently addresses reviewer’s concerns.
